# Natural Killer Cell Dysfunction Causes Eosinophil Accumulation in Chronic Rhinosinusitis With Nasal Polyps

**DOI:** 10.1002/clt2.70156

**Published:** 2026-02-17

**Authors:** Yohei Sato, Daiki Nakashima, Natsuki Inoue, Erika Osada, Tomomitsu Hirota, Yasuhiro Tsunemi, Nobuyoshi Otori, Mamoru Yoshikawa, Mayumi Tamari, Tsuguhisa Nakayama

**Affiliations:** ^1^ Laboratory of Immune Cell Therapy, Project Research Unit The Jikei University School of Medicine Tokyo Japan; ^2^ Core Research Facilities The Jikei University School of Medicine Tokyo Japan; ^3^ Immunology and Allergy Research Unit, Division of Otorhinolaryngology, Head & Neck Surgery, Faculty of Medicine University of Fukui Fukui Japan; ^4^ Division of Molecular Genetics The Jikei University School of Medicine Tokyo Japan; ^5^ Department of Otorhinolaryngology The Jikei University School of Medicine Tokyo Japan; ^6^ Department of Otorhinolaryngology Toho University Ohashi Medical Center Tokyo Japan; ^7^ Department of Otorhinolaryngology, Head and Neck Surgery Dokkyo Medical University School of Medicine Mibu Tochigi Japan

To the Editor

Chronic rhinosinusitis with nasal polyps (CRSwNP), a chronic nasal and sinonasal inflammatory disease, is characterized by nasal obstruction, nasal discharge, and olfactory disturbances persisting for more than 12 weeks [1]. Notably, although CRSwNP is predominantly characterized as a type 2 inflammation, it may involve mixed inflammatory endotypes, including type 1 and 3 inflammation, with notable regional differences in endotype composition [2]. Whereas natural killer (NK) cell dysfunction may contribute to eosinophil proliferation in CRSwNP, the lack of immune phenotype and gene expression profiles of nasal polyp (NP)‐derived NK cells currently limits further characterization [3, 4].

Recently, NK cells have been considered regulators of allergic reactions in atopic dermatitis [5], and transcriptomic profiles of atopic skin‐derived immune cells have revealed a distinct subtype of atopic dermatitis based on eosinophil infiltration, with dual blockade of interleukin (IL)‐4 and IL‐13 enhancing NK cell signatures [6]. Furthermore, the innate NK–eosinophil immune crosstalk has shown that NK cells can suppress eosinophils through NKp46 and NKp30 upon activation [7]. Reportedly, CRSwNP‐derived NK cells exhibit reduced expression of functional receptors [8]. Herein, we performed transcriptomic analysis and immune cell profiling to investigate the interactions between NK cells and eosinophils, focusing in particular on NK cell phenotype and function. Furthermore, to assess the eosinophil apoptosis induction by NK cells, we co‐cultured these two cell types.

Collagenase digestion was used to isolate immune cells from freshly harvested surgical NP samples of multiple donors (*n* = 26), and cells were sorted and analyzed by flow cytometry (Figure 1A–C; Supporting Information S1: Figure S1; Supporting Information S2: Table S1). Through unbiased clustering, eosinophil‐high (Eos^high^)‐ and eosinophil‐low (Eos^low^)‐infiltrated NPs were grouped, with the eosinophil vector being the predominant factor (Figure 1D,E). NP subtype was determined based on eosinophil percentage (Eos^high^‐infiltrated NPs: 36% ± 9%, Eos^low^‐infiltrated NPs: 8% ± 6%; *p* < 0.0001), and it was found that the percentages of other immune cells were reduced in Eos^high^‐infiltrated NPs (Figure 1F). Whereas the proportions of T‐cells, NK cells, and neutrophils significantly reduced, the proportions of B‐cells or monocytes in Eos^high^‐infiltrated NPs did not significantly reduce (Supporting Information S1: Figure S2). Moreover, we detected notable differences in the whole‐transcriptome profiles of NK cells, which differed in Eos^high^‐ and Eos^low^‐infiltrated NPs (Figure 2A). Interestingly, compared with those in Eos^low^‐infiltrated NPs, Eos^high^‐infiltrated NPs promoted an upregulation of type 2 inflammatory markers and downregulation of NK receptors and cytotoxic molecules (Figure 2B). Reportedly, the expression of type 2 inflammatory markers can be induced in NK cells via signal transducer and activator of transcription six expression [9].

**FIGURE 1:**
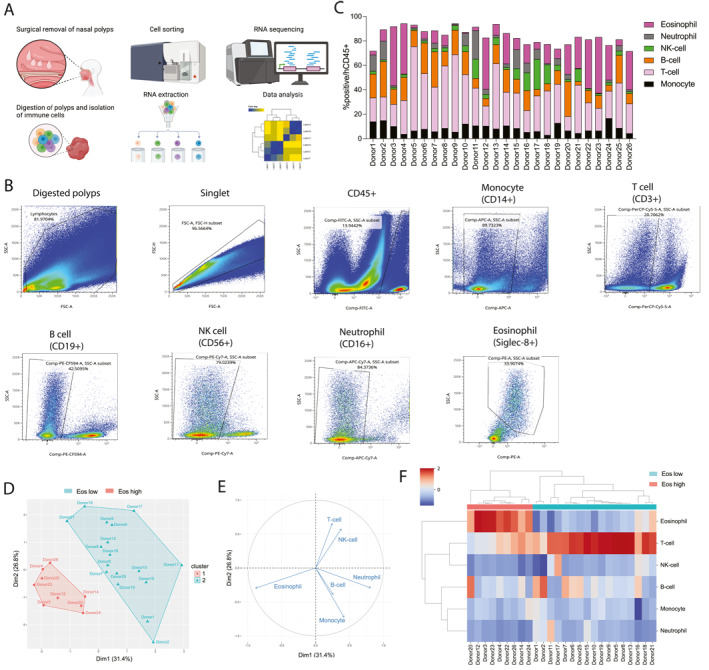
Immunophenotype of chronic rhinosinusitis with nasal polyps analyzed using flow cytometry. (A) Study design. (B) Gating strategy of immune cells isolated from nasal polyps. (C) Immunophenotype of CD45+ cells isolated from polyps (*n* = 26). Distribution of immune cells indicated by the results of principal component analysis‐based clustering (D) and vector (E) of each cell type (*n* = 26). (F) Lineage distribution presented in a heatmap with clustering (*n* = 26). CD, cluster of differentiation; Eo^high^, eosinophil‐high; Eo^low^, eosinophil‐low; NK, natural killer.

**FIGURE 2:**
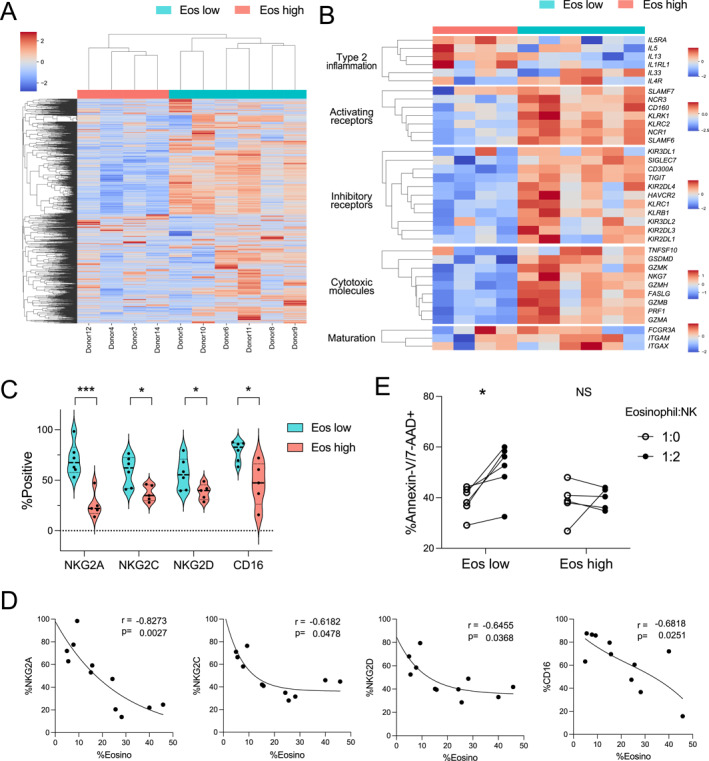
NK cells isolated from Eos^high^‐infiltrated polyps showed downregulation of NK receptors. (A) Gene expression profile and (B) targeted gene expression of NK cells isolated from Eos^high^‐infiltrated polyps (*n* = 10). (C) Immunophenotype of NK cells isolated from chronic rhinosinusitis with nasal polyps analyzed by flow cytometry and compared with the frequency of eosinophils. *p < 0.05, ***p < 0.001, Unpaired *t*‐test. (D) Spearman's correlation analysis of the association between the frequency of eosinophils (*x*‐axis) and the expressions of NK receptors (*y*‐axis) of all polyp donors (*n* = 11). (E) The frequency of apoptotic eosinophils co‐cultured with and without NK cells at Eosinophil: NK cell ratios of 1:0 (eosinophil only) and 1:2 (co‐culture). Polyps with Eos^low^ (*n* = 6) and Eos^high^ (*n* = 5) infiltration were analyzed independently. *p < 0.05, ***p < 0.001, Paired *t*‐test. CD, cluster of differentiation; Eos^high^, eosinophil‐high; Eos^low^, eosinophil‐low; NK, natural killer; NKG2A, NK Group 2 member A; NKG2C, NK Group 2 member C; NKG2D, NK Group 2 member D. NS; not significant.

Based on the transcriptomic analysis, we hypothesize that the downregulation of NK receptors may enhance eosinophil proliferation in CRSwNP. The properties of NK cells were further delineated based on the analysis of the inhibitory NK Group 2 member A (NKG2A) and activating NK Group 2 member C (NKG2C) and NK Group 2 member D (NKG2D), and low‐affinity IgG Fc (cluster of differentiation [CD]16) receptors. The flow cytometric results revealed a number of differentially expressed proteins, including inhibitory, activating, and low‐affinity IgG Fc receptors (Supporting Information S1: Figure S3). The levels of NKG2A, NKG2C, NKG2D, and CD16 were markedly reduced in Eo^high^‐infiltrated NPs compared with those in Eo^low^‐infiltrated NPs (Figure 2C). Moreover, the levels of NKG2A/2C/2D and CD16 were found to be negatively correlated with eosinophil percentage in NPs (Figure 2D).

To assess NK cell‐mediated cytotoxicity, we co‐cultured NK cells and eosinophils derived from the same NPs (Supporting Information S1: Figure S4). Owing to the limited viability of eosinophils isolated by flow cytometry (< 30%), eosinophils and NK cells were isolated from NPs via magnetic isolation by targeting Siglec‐8 (eosinophil) and CD56 (NK cells). Interestingly, whereas Eo^high^‐infiltrated NP‐derived NK cells had no significant effects on the viability of co‐cultured eosinophils, those isolated from Eo^low^‐infiltrated NPs were found to cause a reduction in eosinophil viability (Figure 2E). These results provide evidence to indicate the functional association of NK cell activity with the classification of CRSwNP per eosinophil infiltration. Hence, NK cell dysfunction in Eo^high^‐infiltrated CRSwNP may promote eosinophil survival and accumulation, potentially exacerbating disease progression and the therapeutic response, owing to immunological alterations (Figure 2C,D).

This study has some limitations. First, eosinophils were isolated by Siglec‐8, which may potentially limit their survival. Second, the number of eosinophils and NK cells freshly isolated from NPs was limited, which hindered conventional NK functional assays, including the K562 killing assay and the degranulation assay. Although RNA isolation from eosinophils was performed, the results were poor. This suggests that eosinophil isolated from severe CRSwNP may exhibit higher resistance to apoptosis, regardless of NK dysfunction.

Overall, this study revealed that NK cell dysfunction due to the downregulated expression of functional molecules results in eosinophil proliferation in NPs derived from patients with CRSwNP. These results provide a basis for gaining a more comprehensive understanding of CRSwNP‐associated immune reactions and for developing potential therapeutic approaches that target the interactions between NK cells and eosinophils.

## Author Contributions

Yohei Sato prepared the manuscript and performed the cell analysis. Daiki Nakashima and Natsuki Inoue were responsible for sample collection and patient enrollment. Erika Osada performed sample preparation and flow cytometry. Tsuguhisa Nakayama designed and supervised the study. All authors contributed to writing the manuscript, and have read and approved the final manuscript.

## Funding

The study was supported by Jikei University School of Medicine.

## Conflicts of Interest

T.N. receives lecture fees and research grants, and SH receives lecture fees from Sanofi. M.T. receives lecture fees from Sanofi, Boehringer Ingelheim, and Astra Zeneca. M.Y. receives lecture fees from Sanofi and contracted research expenses from Kissei Pharmaceutical. The funding sources played no role in the design, conduct, preparation, or writing of this manuscript. The other study authors have no conflicts of interest to disclose.

## Supporting information


Supporting Information S1

Supporting Information S2

## Data Availability

The data that support the findings of this study are available on request from the corresponding author. The data are not publicly available due to privacy or ethical restrictions.
