# Supplementary material for: Natural Killer Cell Dysfunction Causes Eosinophil Accumulation in Chronic Rhinosinusitis With Nasal Polyps
Source: Clin Transl Allergy. 2026 Feb 17;16(2):e70156. doi: 10.1002/clt2.70156 (PMC12912943; doi:10.1002/clt2.70156)
Supplement: Supplementary file 1 — Supporting Information S1 [file CLT2-16-e70156-s001.pdf]

Online Supplementary Material

Online Methods

Study subjects

The study protocol was approved by the Institutional Review Board of Jikei University School of Medicine (#33-407[11032]). For the purposes of this study, we enrolled individuals aged ≥18 years, who had undergone endoscopic sinus surgery between June 2022 and December 2024. The patients were verbally informed of the purpose of the study, and written informed consent was obtained. The diagnosis of chronic rhinosinusitis with nasal polyps (CRSwNP) (1) and surgical indications were determined based endoscopy and computed tomography (CT) examinations, which confirmed the failure of conservative treatment to achieve healing. Nasal polyp (NP) scores were evaluated unilaterally on a scale of 0–4 points (0–8 points bilaterally) (2), whereas CT scores were assessed unilaterally on a scale of 0–12 points (0–24 points bilaterally) (3). A score of 0 indicated the absence of lesions in both assessments. The exclusion criteria were as follows: a history of systemic steroid use within 1 month prior to surgery, use of immunosuppressive medications, application of biological agents within 6 months prior to surgery, and ongoing treatment for malignancy. The JESREC scoring criteria for diagnosing ECRS is a scoring system that assesses unilateral or bilateral disease, the presence of NPs, ethmoid-dominant CT shadows, and eosinophil ratio in the peripheral blood. ECRS was defined as a total score of >11.

Polyp isolation

NPs were surgically removed from patients with CRSwNP and collected in normal saline. Immediately after surgery, mucosal tissues were digested with 1 mg/mL Liberase TL (Roche) dissolved in Roswell Park Memorial Institute (RPMI) medium (Thermo Fisher Scientific) at

37°C for 30–60 min. The digested mucosal tissues were subsequently resuspended in phosphate-buffered saline (PBS) and filtered using a 70-μm filter (Falcon), followed by immediate use for flow cytometry and magnetic cell isolation.

Flow cytometric fluorescence-activated cell sorting (FACS)

Digested mucosal tissues were resuspended in FACS buffer (PBS supplemented with 0.5% bovine serum albumin and 2 mM ethylenediaminetetraacetic acid) and blocked with FcBlock (BD Biosciences) for 10 min. Following Fc blocking, antibody cocktails (Panel 1: Immunophenotype/Sorting, Panel 2: NK phenotype 1, and Panel 3: NK phenotype 2) were incubated with samples at 4°C for 30 min. For Panel 3, the stained cells were fixed with BD Cytofix Fixation Buffer, followed by intracellular staining. Having thereafter centrifuged all stained samples, they were filtered using a cell strainer (Falcon). Data were acquired using a FACSAria III cytometer (BD Biosciences) and analyzed using FlowJo software (ver. 11.0; FlowJo LLC). The gating strategy (Figure 1B) was preliminarily determined using peripheral blood mononuclear cells (PBMCs) and confirmed using tissue samples. The antibodies used are listed in Table S2.

Magnetic-activated cell sorting of immune cells from the tissue

PBMCs and mucosal tissues were stained with phycoerythrin (PE)-conjugated anti-Siglec-8 antibodies. Samples were washed three times with FACS buffer, and the cells were stained with MojoSort Human anti-PE Nanobeads (BD Biosciences) at 4°C for 30 min. Thereafter, Siglec-8-positive cells were magnetically isolated using an EasySep Magnet (StemCell Technologies) in accordance with the manufacturer's protocol. For NK cells, cluster of differentiation (CD)56+ cells were stained using MojoSort anti-CD56 Nanobeads (BD Biosciences) and magnetically isolated using an EasySep Magnet following multiple washes. Isolated cells were washed twice

with FACS buffer and resuspended in RPMI medium (Thermo Fisher Scientific) supplemented with 10% fetal bovine serum (Gibco) and 1% penicillin–streptomycin (Gibco), and were counted using a Countess3 Cell Counter (Thermo Fisher Scientific) for determining cell viability and numbers for the following assays.

Co-culture of NK cells and eosinophils

To evaluate the cytotoxicity of NK cells isolated from NPs, magnetically isolated eosinophils (10,000 cells) and NK cells (20,000 cells) were co-cultured in 96-well U-bottom plates at 37°C for 4–6 h. After incubation, the cells were centrifuged and stained with viability dyes (Annexin-V and 7-AAD) to evaluate cytotoxicity, together with the surface markers of eosinophils (Siglec-8) and NK cells (CD3 and CD56). The percentage of dead cells was calculated in Siglec-8-positive eosinophil populations.

RNA isolation

Total RNA was extracted from 1×10^5 cells using an RNeasy Micro Kit (QIAGEN, Hilden, Germany). Cells were lysed used RNA lysis buffer (QIAGEN), and total cellular RNA was extracted following the manufacturer's protocol. The concentration of the extracted total RNA was measured using a Nanodrop spectrophotometer and/or Qubit HS RNA kit, and the quantified samples were stored at -80°C and sent to a commercial vendor for RNA sequencing (RNA-seq).

RNA-seq

For RNA-seq analysis, the quantity and quality of the purified RNA were initially assessed using an Agilent 2100 Bioanalyzer. Complementary DNA was generated using SMART-seq HT kits (Takara Bio), and sequencing libraries were prepared using a Nextera DNA library Preparation kit. The resulting libraries were quantified using a Qubit kit and assessed for quality using the aforementioned Bioanalyzer. After molecular barcoding, libraries were pooled at equimolar

concentrations and sequenced at 2 × 150 cycles in an Illumina NovaSeq 6000 sequencer, yielding approximately 26 million 150-bp paired-end sequence reads per sample. RNA-seq was performed according to the manufacturer's instructions. Low-quality bases and adapter sequences were trimmed from the raw reads using Trimmomatic v0.39, and read quality was assessed using FastQC v0.11.9. The trimmed reads were subsequently aligned to the human reference genome (GRCh38.p13) using HISAT2 v2.2.1. In addition, transcript assembly and quantification were performed using StringTie v2.1.7 and GENCODE v43 annotation. Only genes with normalized counts ≥10 in at least two samples were retained, whereas genes not meeting this threshold were eliminated to remove low-abundance transcripts. Analysis of differential gene expression was performed using the DESeq2 v1.28.1 package in R.

Statistical analyses

For statistical analyses, we used GraphPad Prism Software (ver. 10.5.0; GraphPad Software, La Jolla, CA, USA). Statistical analyses were performed using unpaired (unpaired sample) and paired (paired sample) *t*-tests. Spearman correlation analysis was performed to evaluate the phenotype of the NK cells. Statistical significance was set at $p < 0.05$.

Supplementary Figures

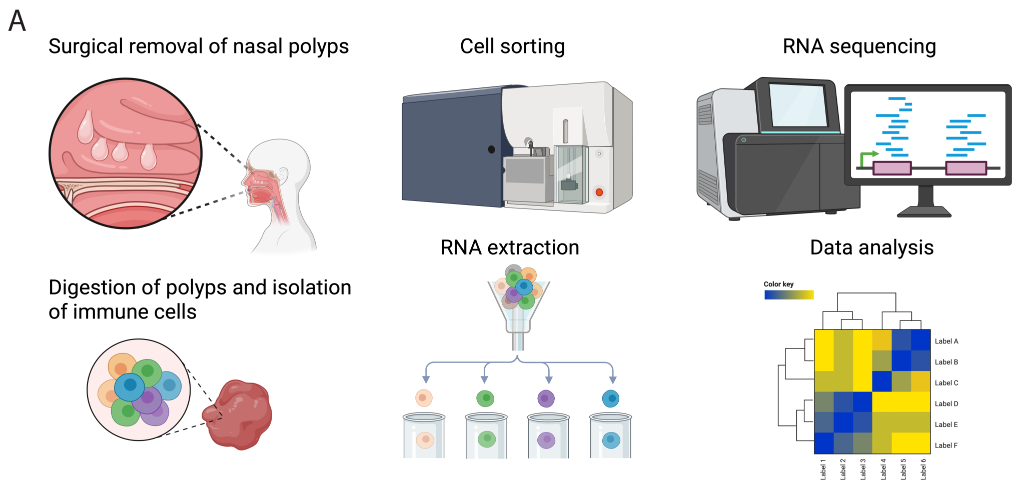

Figure S1. Immunophenotype of chronic rhinosinusitis with nasal polyps. (A) Experimental design.

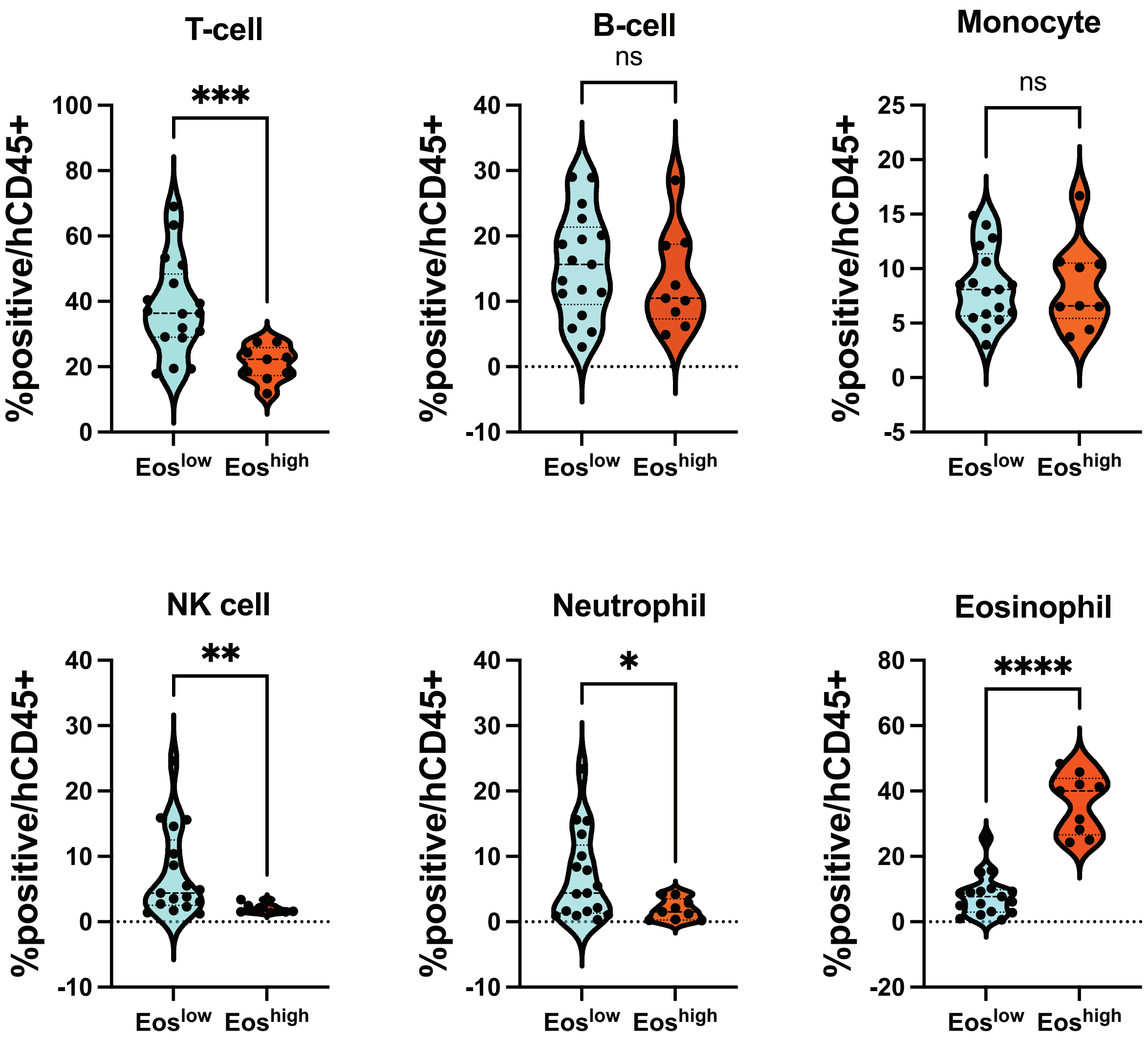

Figure S2. Proportions of immune cell populations. The relative proportions of immune cell populations were determined using fluorescence-activated cell sorting. Eo^{high}, eosinophil-high; Eo^{low}, eosinophil-low.

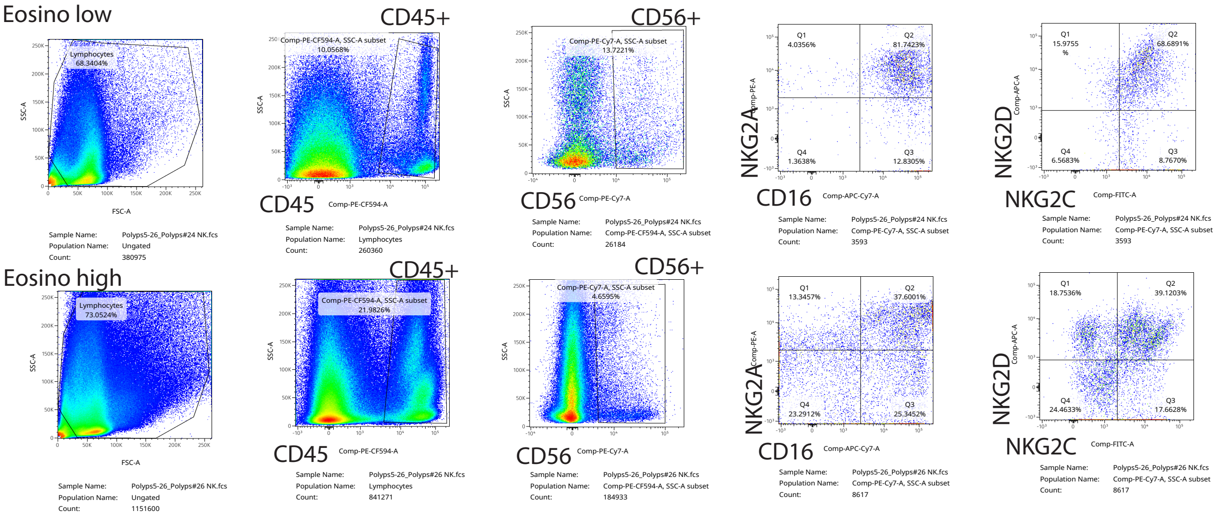

Figure S3. The gating strategy used for natural killer cell phenotypes. Expression of CD16, NKG2A, NKG2C, and NKG2D was determined using fluorescence-activated cell sorting. NKG2A, natural killer group 2 member A; NKG2C, natural killer group 2 member C; NKG2D, natural killer group 2 member D; CD, cluster of differentiation.

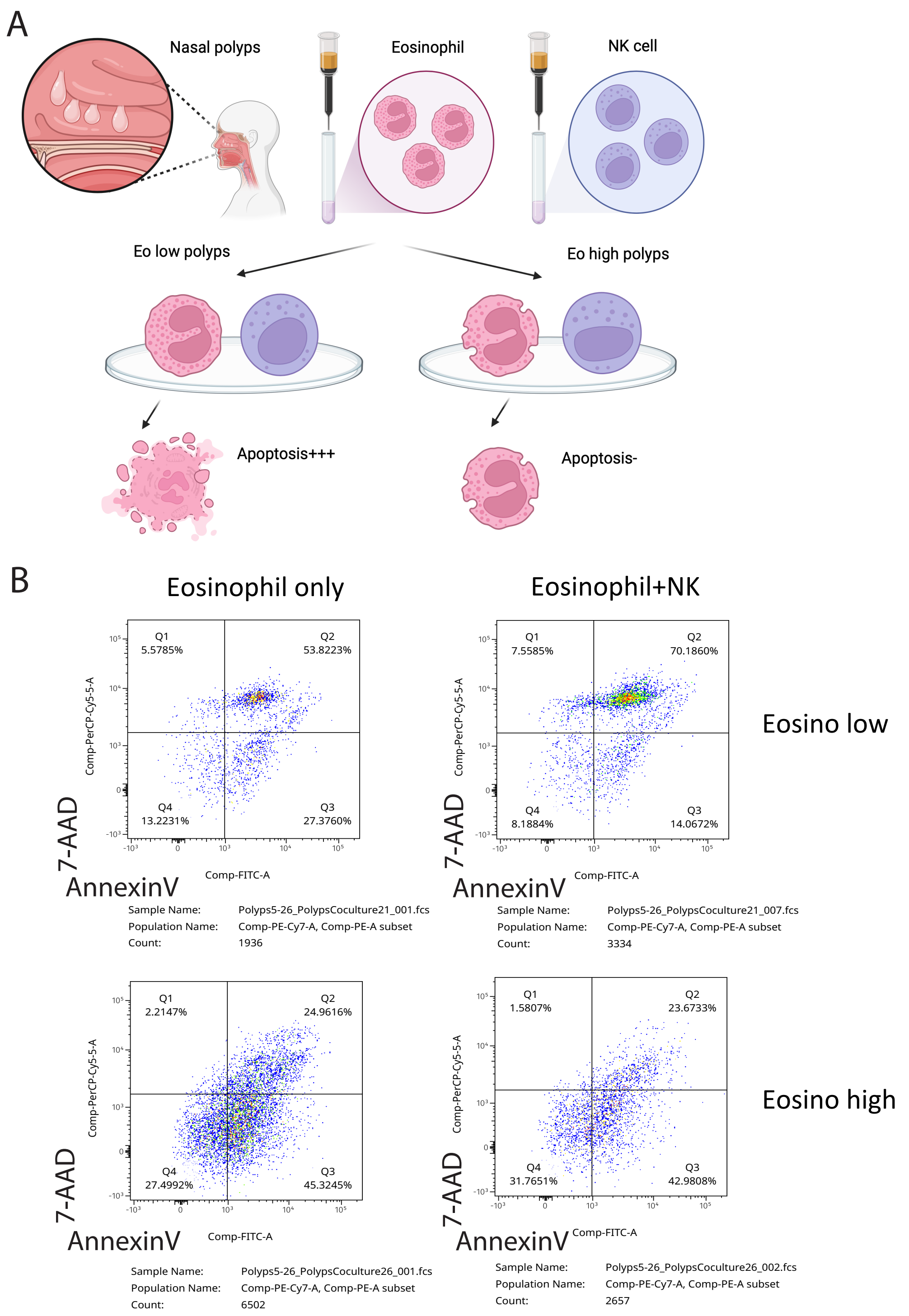

Figure S4. The co-culture of eosinophils and NK cells. (A) Experimental design. (B) Gating strategy used for eosinophils after incubation for 4–6 h with and without NK cells. NK, natural killer; Eo^{high}, eosinophil-high; Eo^{low}, eosinophil-low.
